# Comparing antibiotic treatment at dry-off on one quarter versus all quarters in cows with only one quarter affected with a high somatic cell count or an intramammary infection

**DOI:** 10.3168/jdsc.2024-0674

**Published:** 2024-12-20

**Authors:** P. Silva Boloña, A. Valldecabres, C. Clabby, P. Dillon

**Affiliations:** 1 Teagasc, Animal & Grassland Research and Innovation Centre, Moorepark, Fermoy, Co. Cork, P61 C302, Ireland; 2 School of Veterinary Medicine, Universidad Cardenal Herrera–CEU, CEU Universities, Valencia, Spain 46115

## Abstract

The aim of this randomized controlled study was to evaluate, on cows with only one quarter affected at dry-off with an IMI, a high SCC (>200,000 cells/mL), or both, the impact of treating with an antibiotic plus internal teat sealant only the quarter affected (treating unaffected quarters with teat sealant alone; SelectAB) versus all quarters (AllAB) on subsequent lactation SCC (analyzed as log_10_ transformed SCC) and IMI (odds of cure [i.e., affected quarter at dry-off but not at calving]; and odds of healthy [i.e., not affected at dry-off and calving]). Ninety-six multiparous cows from 3 research herds were randomly assigned to SelectAB or AllAB. Linear and logistic regression mixed models were used for data analysis. Cows assigned to AllAB had a 0.15 units lower log_10_ SCC (95% CI = −0.19 to −0.11 log points) through the full subsequent lactation compared with SelectAB cows. The odds of cure for an affected quarter were similar for AllAB and SelectAB cows (odds ratio = 0.77; 95% CI = 0.27–2.20), but cows under AllAB had higher odds of having a healthy quarter shortly after calving compared with SelectAB cows (odds ratio = 2.86; 95% CI = 1.21–6.73). In conclusion, a reduced rate of healthy quarters (i.e., increased new infections any time from dry-off to shortly after calving), especially in quarters treated with internal teat sealant alone within an udder with one quarter affected with IMI, high SCC, or both at dry-off, may be a major barrier for the implementation of a quarter-based dry-off treatment approach.

## Highlights

• We compared antibiotic dry-off treatment at the cow versus quarter level.
• Quarter-level treatment resulted in higher SCC in the full new lactation.
• Quarter-level treatment led to higher odds of new infections.
• No differences between treatments (cow or quarter) were found in the odds of a cured quarter.

## Graphical Abstract

**Figure:**
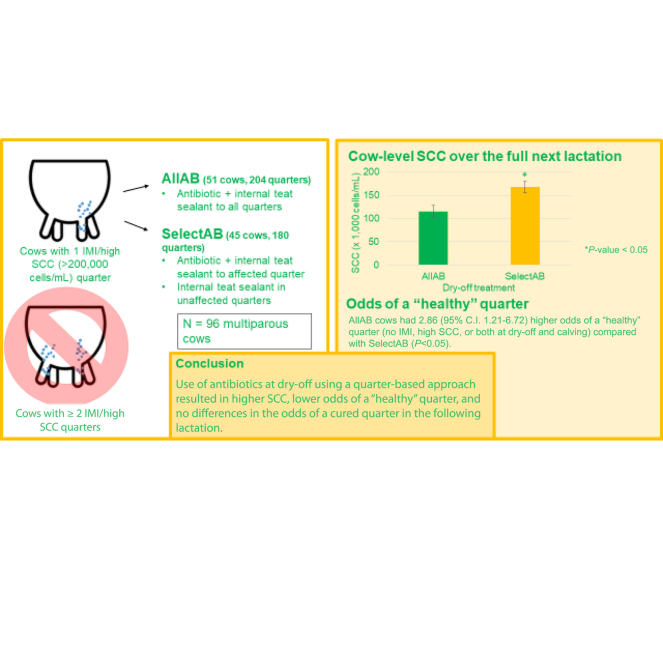
**Summary:** This study compared the effect of targeting dry-off antibiotic treatment to the 1 quarter affected with intramammary infection (IMI) or high somatic cell count (SCC; unaffected quarters were infused with an internal teat sealant [SelectAB: n = 45]) versus treating all quarters (AllAB: n = 51) on SCC and IMI in the new lactation. SelectAB cows had higher SCC in the entire new lactation compared with AllAB cows. There were no differences in the odds of a cured quarter, but SelectAB cows had lower odds of having a quarter not affected with an IMI or a high SCC at dry-off and calving. Error bars in the figure are SE.

In dairy farming, mastitis accounts for the largest use of antibiotics (Pol and Ruegg, 2007), of which antibiotic therapy at dry-off represents its largest use. Treating all quarters of cows with an intramammary antibiotic at the end of lactation has been an important tool used for mastitis control by allowing to cure existing infections (which are less likely to cure when treated in lactation) and to prevent new infections over the dry period (Bradley and Green, 2004). However, due to concerns about antimicrobial resistance, the European Union has set a target for a 50% reduction in antimicrobial sales for animal production systems by 2030 (European Commission, 2020). Additionally, regulation 2019/6 on veterinary medicines by the European Union (European Parliament and the Council of the European Union, 2019) states that antibiotics as a preventative measure should not be used in groups of animals, and therefore treating all cows at dry-off with intramammary antibiotics is no longer a tolerable practice. The use of an internal teat sealant has proven to be effective in preventing new infections over the dry period in cows uninfected at dry-off (Huxley et al., 2002; Bradley et al., 2010). The scientific literature has mostly shown no impact on SCC by treating uninfected cows with internal teat sealant alone compared with teat sealant plus antibiotic (Bradley et al., 2010; Vasquez et al., 2018). However, studies in Ireland have shown a negative impact of using teat sealant alone at dry-off on SCC compared with antibiotic plus teat sealant (McParland et al., 2019; Clabby et al., 2022). Possible reasons for these different results are that in the studies conducted in Ireland herd-level infections were high and that most infections were caused by *Staphylococcus aureus*, a contagious bacterium.

An alternative to reduce the use of antibiotics at dry-off is to treat only the quarters identified as infected while the remaining quarters are not treated at all or are infused with an internal teat sealant alone. Kabera et al. (2020) compared the impact on intramammary infections and SCC between treating only infected quarters with antibiotic plus teat sealant (assessed by on-farm culture), while the remaining quarters were treated with teat sealant alone compared with treating all quarters with antibiotic plus teat sealant. They found no significant differences on new infections, persistence of infections, or SCS between the 2 different approaches (Kabera et al., 2020). In contrast, Browning et al. (1990) found that treating only infected quarters resulted in almost 4 times greater rates of new intramammary infections in the dry period compared with treating all quarters. However, noninfected quarters were not treated with an internal teat sealant in this study. For these 2 studies, there was no distinction of treatment if a cow had one or more than one infected quarter. It has been reported that the number of quarters infected has an influence on the probability of cure, especially when these infections are caused by *S. aureus* (Sol et al., 1997; Barkema et al., 2006).

Approaching dry cow therapy decisions accounting for the quarter infection status is justified given that new regulations imply that evidence is needed to prescribe antibiotic treatment. Selecting antibiotic dry-off treatment at the quarter level would constitute an important reduction in antibiotic use, given that most cows will not have an infection in all 4 quarters. However, in addition to the reduction in the use of antimicrobials, the success of the dry-off management needs to consider the absence of new infections and the elimination of existing infections (Kabera et al., 2020). We hypothesized that following a protocol to treat only the quarter affected with an IMI, a high SCC, or both, we would be able to target antibiotic treatment at dry-off, curing the affected quarters without risk of increasing new IMI or SCC in the uninfected quarters. Therefore, the aim of this study was to evaluate the impact of treating only the affected quarter with antibiotic plus internal teat sealant (while unaffected quarters were treated with teat sealant alone) versus antibiotic plus internal teat sealant in all quarters in cows with only one quarter affected with an IMI, a high SCC, or both at dry-off on subsequent lactation IMI and SCC.

The study was conducted between November 2019 and December 2020 in 3 research dairy herds in the Republic of Ireland (Teagasc Moorepark [herd size = 260], Teagasc Curtins [herd size = 160], and Clonakilty Agricultural College [herd size = 190]). These herds are spring-calving pasture-based herds, where the majority of cows calve between February and April and are dried off between November and December. During the dry period cows are housed in covered cubicle sheds with rubber mats. The cubicles are scraped and disinfected with lime once per day. Cows calve in individual straw bedded calving pens. Upon calving and depending on weather, cows are turned out to grass and milked twice a day. These herds conducted weekly milk recordings to determine milk SCC, yield, and components. Most cows in the study were Holstein Friesians, whereas a minority also had Jersey, Norwegian Red, and Ayrshire genetics. Udder health practices in the study herds involved clinical mastitis diagnosis, based on observable abnormalities in the milk or the udder and subsequent treatment with intramammary antibiotic. As well as, weekly individual cow SCC determination and aseptic quarter sampling for laboratorial culture when a high SCC (≥200,000 cells/mL) was detected to support farms' managers decision to treat, dry quarter, or do nothing. Average bulk tank SCC (cells/mL) in 2019 was 214,000, 174,000, and 103,000 for the Moorepark, Curtins, and Clonakilty herds, respectively.

Before dry-off (average 3 d, range 1–7 d), researchers collected aseptic quarter milk samples (from here onward “dry-off quarter samples”) to assess bacterial IMI and determine quarter-level SCC by flow cytometry (Fossomatic, Foss, Hiller⊘d, Denmark) as described by Clabby et al. (2022). In short, samples were plated in a nonselective medium Blood Agar Base No. 2 (Oxoid) with 0.1% esculin to improve differentiation between *Streptococcus* spp. The plates were divided into 4 equal quadrants (one for each quarter of the same cow) and samples were plated using one 10-μL aseptic disposable loop per quarter. Plates were then incubated at 37°C and bacterial colony morphology was assessed 24 to 48 h after incubation. According to bacterial growth, quarters were classified as having an IMI if at least 6 cfu of the same pathogen was observed in the plated quadrant (McParland et al., 2019; Clabby et al., 2022). According to milk SCC, quarters were classified as high SCC if they had ≥200,000 cells/mL. A sample was considered contaminated if >2 different types of colonies grew on the same quadrant of the plate; no samples had ≥2 types of colonies at dry-off, whereas 6 calving samples from 2 cows were considered contaminated. All cows with 4 functional quarters that had only one quarter affected by an IMI, a high SCC, or both and that were due to remain in the research herds for the following lactation were eligible in the study. The estimated number of cows needed to detect a 10% difference in log_10_ transformed SCC with an average of 4.6 and an SD 0.8, an α level of 0.05, and 0.8 power, was 41 cows per treatment group (Snedecor and Cochran, 1989).

Using the IMI and SCC results from dry-off quarter milk samples, eligible cows were randomly assigned (RAND function in Excel, 2016, Microsoft, Redmond, WA) to receive 1 of 2 dry-off treatments: infusion of an intramammary antibiotic plus an internal teat sealant in all quarters (**AllAB**), or infusion of an intramammary antibiotic plus an internal teat sealant in the affected quarter (i.e., quarter with IMI, high SCC, or both) and internal teat sealant alone in the unaffected quarters (**SelectAB**). All eligible cows were enrolled in the study. The antibiotic infused was cefalonium (Cepravin Dry Cow, MSD, Merck & Co. Inc., Rahway, NJ). Dry-off was conducted by research and farm staff; no blinding was possible given the nature of the study treatments. However, farm staff were uninvolved in the random treatment assignment process.

After calving (4 to 7 DIM), aseptic quarter samples (“calving quarter samples” from here onward) were collected again for bacterial IMI and SCC determination and health status classification as described previously. According to dry-off and calving quarter samples results quarters were classified as **NewIMISCC**, unaffected at dry-off (without an IMI [i.e., pathogen free] and without a high SCC [i.e., ≤200,000 cells/mL]) and affected (with an IMI or a high SCC, or both) at calving. **PersistentIMISCC**, affected at dry-off and calving (in the case of an IMI, affected with the same pathogen species recovered from the dry-off and calving quarter sample). **CuredIMISCC**, affected at dry-off and unaffected at calving (i.e., pathogen free and SCC ≤200,000 cells/mL). **Healthy**, unaffected at dry-off and calving (i.e., pathogen free and SCC ≤200,000 cells/mL).

The primary objective of the statistical analysis was to assess the impact of dry-off treatment (AllAB or SelectAB) on SCC at the first test-day and the full lactation. The secondary objective was to analyze the impact of dry-off treatment on the odds of a CureIMISCC (as opposed to a PersistentIMISCC quarter) and the odds of a Healthy quarter (as opposed to a NewIMISCC quarter). Log_10_ transformation was performed on SCC data before statistical analysis to address the highly skewed nature of this data (log_10_ SCC from here onward). Cow-level SCC data were available on a weekly basis for the 2020 lactation as a result of herd's milk recordings. Cow parity, dry-off, and calving date information were obtained from farm records.

Statistical analysis was performed in SAS (SAS 9.4, SAS Institute Inc., Cary, NC). Multiple linear regression mixed models (MIXED procedure) were used to assess the impact of dry-off treatment (AllAB or SelectAB) on SCC at the first test-day and the full lactation. The models included treatment (AllAB or SelectAB) as fixed effect, and parity (2, 3, or ≥4), SCC at previous lactation's last milk recording (last SCC), DIM, and milk yield at each milk recording as covariates. The random effects of farm and cow within farm were respectively included in the models for SCC at the first test-day and SCC in the full lactation, the last with a compound symmetry covariance structure. Cows with <10 test-days over the lactation (n = 3) were excluded from the full lactation analysis. To provide untransformed LSM estimates, the same mixed models were also analyzed using raw SCC values; *P*-values correspond to the models using log-transformed data. Logistic regression (GENMOD procedure) was used to analyze the impact of dry-off treatment on the odds of a CureIMISCC or a Healthy quarter after calving. The models included the fixed effect of treatment and parity (2, 3, or ≥4) as a covariate and the random effect of cow to account for the correlation between quarters within each cow.

In total, 518 cows were sampled at dry-off in 2019. Of those, 38% (199/518) did not have affected quarters, 27% (138/518) had only one quarter with a high SCC, an IMI, or both and were eligible for the study, and 35% (181/518) had >1 quarter affected. Among cows only with IMI, 65.3% (124/190) had one quarter and 34.7% (66/190) had >1 quarter affected. After excluding cows with <4 functional quarters and due to leave the herd, 96 cows fulfilled the eligibility criteria. These were randomly assigned to SelectAB (n = 45) or AllAB (n = 51). Cows had one quarter with high SCC (AllAB: n = 20; SelectAB: n = 23), IMI (AllAB: n = 10; SelectAB: n = 6), or both (AllAB: n = 21; SelectAB: n = 16). In total, 204 intramammary antibiotic tubes were used in the 51 AllAB cows, whereas 45 antibiotic tubes were used for the 45 SelectAB cows.

Six calving quarter milk samples from 2 cows (one in each treatment) were contaminated and not used for the logistic regression analysis. Three cows were culled with <10 test-days (SelectAB: n = 2, AllAB: n = 1); their data were used for the logistic regression analysis but were not used for the full lactation mixed model analysis. Table 1 describes cow-level information, milk yield, and SCC by treatment for study cows.

**Table 1:** Cow-level information, milk yield, and SCC at dry-off 2019 and at first test-day of the 2020 lactation (n = 96 cows)

Variable by time	Treatment^1^	Mean	SD	Minimum	Maximum
Dry-off					
SCC, cells/mL	AllAB	168,000	208,000	17,800	1,300,000
	SelectAB	200,000	229,000	31,000	1,100,000
DIM at dry-off, d	AllAB	285	18	224	331
	SelectAB	284	20	229	311
Days dry, d	AllAB	76	16	52	122
	SelectAB	80	17	51	120
First test-day					
DIM, d	AllAB	8.1	3.0	4	17
	SelectAB	9.3	4.3	4	22
Parity	AllAB	3.7	1.7	2	8
	SelectAB	3.9	2.0	2	9
Milk yield, kg	AllAB	26.5	4.5	9.2	38.1
	SelectAB	26.1	3.7	19.2	33.8
SCC, cells/mL	AllAB	147,400	249,200	7,000	1,300,000
	SelectAB	157,200	304,400	3,000	1,500,000

1 AllAB: all quarters treated with an intramammary antibiotic plus internal teat sealant; SelectAB: only the quarter with IMI or high SCC (>200,000 cells/mL), or both, was treated with an intramammary antibiotic and an internal teat sealant, whereas the other quarters were treated with an internal teat sealant alone.

Of the affected quarters at dry-off, 94.1% (48/51; AllAB) and 95.6% (43/45; SelectAB) cured and were unaffected at calving (CuredIMISCC), whereas 5.9% (3/51; AllAB) and 4.5% (2/45; SelectAB) were still affected at calving (PersistentIMISCC). Of the unaffected quarters at dry-off, 96.0% (141/147; AllAB) and 88.9% (120/135; SelectAB) were still unaffected at calving (Healthy), whereas 4.0% (6/147; AllAB) and 11.1% (15/135; SelectAB) were affected at calving (NewIMISCC). Dry-off quarter-level IMI (n = 53) were caused by *S. aureus* (n = 52) and *Streptococcus uberis* (n = 1). Calving quarter-level IMI (n = 10) were caused by *S. aureus* (n = 8), *Strep. uberis* (n = 1), and *Escherichia coli* (n = 1).

Average log_10_ SCC at the first milk recording and full lactation were 4.78 and 5.10, respectively. Dry-off treatment was not associated with log_10_ SCC at the first test-day of the new lactation (*P* = 0.79), while accounting for the effects of parity and previous lactation's last SCC (Table 2). In contrast, when looking at the full lactation, dry-off treatment was associated with a 0.15 units greater log_10_ SCC throughout lactation (*P* < 0.001) for SelectAB cows compared with AllAB cows while accounting for the effects of parity and previous lactation's last SCC (Table 2). Treatment was not associated with the odds of a CureIMISCC quarter (Table 3). In contrast, AllAB cows had 2.9 higher odds of having a Healthy quarter compared with SelectAB cows (Table 3).

**Table 2:** Mixed model analysis of the impact of dry-off treatment on log_10_ SCC at the first milk recording and the full lactation (n = 95^1^)

Parameter	Estimate (untransformed SCC^2^)	95% CI	*P*-value
First milk recording			
Treatment^3^			
AllAB	−0.03 (950)	−0.44 to 0.51	0.79
SelectAB	Referent		
Parity			
2	−0.01 (−11,700)	−0.38 to 0.36	0.94
3	−0.27 (−108,000)	−0.67 to 0.13	0.13
≥4	Referent		
LastSCC,^4^ cells/mL	−0.0002 (9)	−0.0004 to 0.0003	0.9
			
Full lactation			
Treatment			
AllAB	−0.15 (−52,000)	−0.19 to −0.11	<0.0001
SelectAB	Referent		
Parity			
2	−0.16 (−90,200)	−0.21 to −0.12	<0.0001
3	−0.11 (−90,500)	−0.16 to −0.06	<0.0001
≥4	Referent		
LastSCC,^4^ cells/mL	0.0002 (380)	0.0001 to 0.0003	<0.0001

1 One cow was sold before its first milk recording.

2 The model was also analyzed with raw SCC (cells/mL) and the untransformed LSM estimates are shown in parentheses.

3 AllAB: all quarters treated with an intramammary antibiotic plus internal teat sealant; SelectAB: only the quarter with IMI or high SCC (>200,000 cells/mL), or both, was treated with an intramammary antibiotic and an internal teat sealant, whereas the other quarters were treated with an internal teat sealant alone.

4 Last milk recording SCC for the 2019 (previous) lactation.

**Table 3:** Odds ratio estimates for cured (quarter with IMI or high SCC [i.e., >200,000 cells/mL] at dry-off and without IMI or high SCC at calving) or healthy (quarter without IMI or high SCC at dry-off and calving) quarters after calving

Parameter by outcome	Odds ratio estimate	95% CI	*P*-value
CuredSCCIMI quarter			
Treatment^1^			
AllAB	0.77	0.27 to 2.20	0.6
SelectAB	Referent		
Parity			
2	2.20	0.90 to 5.40	0.08
3	1.27	0.74 to 2.20	0.4
≥4	Referent		
			
Healthy quarter			
Treatment^1^			
AllAB	2.86	1.21 to 6.73	0.01
Select AB	Referent		
Parity			
2	0.41	0.25 to 0.67	0.0004
3	1.33	1.02 to 1.74	0.03
≥4	Referent		

1 AllAB: all quarters treated with an intramammary antibiotic plus internal teat sealant; SelectAB: only the quarter with IMI or high SCC (>200,000 cells/mL), or both, was treated with an intramammary antibiotic and an internal teat sealant, whereas unaffected quarters were infused with an internal teat sealant alone.

In agreement with our findings, previous research treating with an intramammary antibiotic at dry-off all quarters or only the infected quarters (leaving the uninfected quarters untreated and without internal teat sealant), found that the rate of new IMI was greater for the group treated in the infected quarters only compared with the group treated with antibiotics in all quarters (Browning et al., 1990), potentially due to the dual function of antibiotics of curing existing infections and preventing new infections (Bradley and Green, 2004). However, they compared protocols rather than treatments, as uninfected and untreated cows were also included in the study groups. In contrast, Kabera et al. (2020) compared a quarter-based approach versus a blanket approach and found no differences in new IMI rates, persistence of IMI or SCS. However, it is unclear in the reporting of that study if there were cows in the quarter-based approach who had all their quarters uninfected and infused with internal teat sealant alone at dry-off. In contrast with our findings, Swinkels et al. (2021) compared the antibiotic treatment of all quarters versus selectively treating quarters based on 2 thresholds of the California Mastitis Test (**CMT**) before dry-off in cows with a SCC >200,000 cells/mL, and showed no differences in the odds of having an infected quarter after calving, but a greater SCC at the first test-day for cows treated at the quarter level. Dohoo (1993) mentioned that reporting SCC soon after calving (within 9 to 11 DIM) could lead to a bias in the estimation of elevated high SCC. Because SCC can be elevated in very early lactation, our lack of differences between the treatment groups at the first milk recording (conducted on average at 8 DIM) may be due to a naturally occurring high SCC in both groups. In these research herds, the average SCC of cows between 8 to 11 DIM was 101,000 cells/mL in 2024. This is supported by our finding that cows in the AllAB group had higher odds of a Healthy quarter after calving compared with SelectAB.

Our study used only cows with one quarter affected with an IMI, a high SCC, or both at dry-off, seeking homogeneity in the sample because cure rates decrease with increasing number of quarters infected (Barkema et al., 2006). According to previous data from our team, in 21 commercial herds, of the cows that had IMI at dry-off, most (76%) had IMI in 1 quarter (with 19% in 2 and 5% in 3 or 4 quarters; Valldecabres et al., 2023). Unfortunately, the number of quarters affected has not been reported in previous studies (Kabera et al., 2020; McDougall et al., 2022). The similar odds of cure between AllAB and SelectAB cows, would support this quarter-based treatment approach; however, the high apparent cure rate (SelectAB: 95.6%; AllAB: 94.0%) was driven by only 2 and 3 quarters treated with intramammary antibiotic that did not cure over the dry period (PersistentIMISCC) for the SelectAB and AllAB treatments, respectively. In the study by McDougall et al. (2022), bacteriological cure rate did not differ between quarters treated with antibiotic plus internal teat sealant at the cow or quarter level (CMT score of ≥trace).

By using a quarter-level approach, antibiotic use can often be reduced. Browning et al. (1990) calculated that by assigning dry cow therapy at the cow level, 696 tubes of antibiotic were used to eliminate 109 IMI compared with a quarter-based approach where 275 tubes were used to eliminate 66 IMI. Using an on-farm culture system for antibiotic dry cow therapy, a greater reduction in antibiotic use is observed when treatment is implemented at the quarter level (58%; Kabera et al., 2020) than at the cow level (22%; Cameron et al., 2014). Conversely, McDougall et al. (2022) found that assigning antibiotic dry cow therapy based on a CMT score of “trace” at the quarter level resulted in lower new IMI and greater antibiotic use compared with assigning antibiotic dry cow treatment to all quarters based on SCC records, but this is most likely due to the differences in the tests used for selection of antibiotic therapy. In our study, there was an obvious reduction in antibiotic use at dry-off with the SelectAB approach because only the affected quarter was treated and the study was designed to include only cows with one quarter affected with IMI, high SCC, or both.

The sensitivity of the tests applied may have conditioned the results found in our study. Our definition of IMI according to the work of Dohoo et al. (2011) for a single quarter sample would have resulted in a sensitivity of 72.4% to 90.4% for *S. aureus*, and 80.8% to 86.5% for *Strep. uberis*, but lower for other minor pathogens. This is the reason we included a high SCC quarter for enrollment in the study. Djabri et al. (2002) described that the SCC geometric mean for quarters infected with *S. aureus* varied between 158,000 and 2,525,000 cells/mL and with *Strep. uberis* between 609,000 and 1,538,000 cells/mL. Lower thresholds for the definitions of IMI and a high SCC should be explored to potentially allow greater sensitivity and identification of minor pathogen IMI.

In conclusion, this study showed that the use of antibiotics at dry-off only on quarters with an IMI, a high SCC, or both resulted in greater SCC, lower odds of a Healthy quarter, and similar odds of a cured quarter in the following lactation, in herds where *S. aureus* was the main pathogen causing IMI. Future research will focus the attention on preventing new infections in the dry period, especially in cows treated with internal teat sealant alone as this seems to be one of the major barriers for implementation of a quarter-based dry-off antibiotic treatment approach.

## References

[bib1] Barkema H.W., Schukken Y.H., Zadoks R.N. (2006). Invited review: The role of cow, pathogen, and treatment regimen in the therapeutic success of bovine *Staphylococcus aureus* mastitis. J. Dairy Sci..

[bib2] Bradley A.J., Breen J.E., Payne B., Williams P., Green M.J. (2010). The use of a cephalonium containing dry cow therapy and an internal teat sealant, both alone and in combination. J. Dairy Sci..

[bib3] Bradley A.J., Green M.J. (2004). The importance of the nonlactating period in the epidemiology of intramammary infection and strategies for prevention. Vet. Clin. North Am. Food Anim. Pract..

[bib4] Browning J.W., Mein G.A., Barton M., Nicholls T.J., Brightling P. (1990). Effects of antibiotic therapy at drying off on mastitis in the dry period and early lactation. Aust. Vet. J..

[bib5] Cameron M., McKenna S.L., MacDonald K.A., Dohoo I.R., Roy J.P., Keefe G.P. (2014). Evaluation of selective dry cow treatment following on-farm culture: Risk of postcalving intramammary infection and clinical mastitis in the subsequent lactation. J. Dairy Sci..

[bib6] Clabby C., McParland S., Dillon P., Arkins S., Flynn J., Murphy J., Silva Boloña P. (2022). Internal teat sealants alone or in combination with antibiotics at dry-off–the effect on udder health in dairy cows in five commercial herds. Animal.

[bib7] Djabri B., Bareille N., Beaudeau F., Seegers H. (2002). Quarter milk somatic cell count in infected dairy cows: A meta-analysis. Vet. Res..

[bib8] Dohoo I.R. (1993). An evaluation of the validity of individual cow somatic cell counts from cows in early lactation. Prev. Vet. Med..

[bib9] Dohoo I.R., Smith J., Andersen S., Kelton D.F., Godden S., Mastitis Research Workers' Conference (2011). Diagnosing intramammary infections: Evaluation of definitions based on a single milk sample. J. Dairy Sci..

[bib10] European Commission (2020). Farm to Fork Strategy: For a Fair, Healthy and Environmentally-Friendly Food System. https://food.ec.europa.eu/system/files/2020-05/f2f_action-plan_2020_strategy-info_en.pdf.

[bib11] European Parliament and the Council of the European Union (2019). Regulation 2019/6 of the European Parliament and of the Council of 11 December 2018 on veterinary medicinal products and repealing Directive 2001/82/EC. Off. J. Eur. Union L.

[bib12] Huxley J.N., Green M.J., Green L.E., Bradley A.J. (2002). Evaluation of the efficacy of an internal teat sealer during the dry period. J. Dairy Sci..

[bib13] Kabera F., Dufour S., Keefe G., Cameron M., Roy J.P. (2020). Evaluation of quarter-based selective dry cow therapy using Petrifilm on-farm milk culture: A randomized controlled trial. J. Dairy Sci..

[bib14] McDougall S., Williamson J., Lacy-Hulbert J. (2022). Bacteriological outcomes following random allocation to quarter-level selection based on California Mastitis Test score or cow-level allocation based on somatic cell count for dry cow therapy. J. Dairy Sci..

[bib15] McParland S., Dillon P.G., Flynn J., Ryan N., Arkins S., Kennedy A. (2019). Effect of using internal teat sealant with or without antibiotic therapy at dry-off on subsequent somatic cell count and milk production. J. Dairy Sci..

[bib16] Pol M., Ruegg P.L. (2007). Treatment practices and quantification of antimicrobial drug usage in conventional and organic dairy farms in Wisconsin. J. Dairy Sci..

[bib17] Snedecor G.W., Cochran W.G. (1989).

[bib18] Sol J., Sampimon O.C., Snoep J.J., Schukken Y.H. (1997). Factors associated with bacteriological cure during lactation after therapy for subclinical mastitis caused by *Staphylococcus aureus.*. J. Dairy Sci..

[bib19] Swinkels J.M., Leach K.A., Breen J.E., Payne B., White V., Green M.J., Bradley A.J. (2021). Randomized controlled field trial comparing quarter and cow level selective dry cow treatment using the California Mastitis Test. J. Dairy Sci..

[bib20] Valldecabres A., Clabby C., Dillon P., Silva Boloña P. (2023). Association between quarter-level milk somatic cell count and intramammary bacterial infection in late-lactation Irish grazing dairy cows. JDS Commun..

[bib21] Vasquez A.K., Nydam D.V., Foditsch C., Wieland M., Lynch R., Eicker S., Virkler P.D. (2018). Use of a culture-independent on-farm algorithm to guide the use of selective dry-cow antibiotic therapy. J. Dairy Sci..

